# A case study of an integrative genomic and experimental therapeutic approach for rare tumors: identification of vulnerabilities in a pediatric poorly differentiated carcinoma

**DOI:** 10.1186/s13073-016-0366-0

**Published:** 2016-10-31

**Authors:** Filemon S. Dela Cruz, Daniel Diolaiti, Andrew T. Turk, Allison R. Rainey, Alberto Ambesi-Impiombato, Stuart J. Andrews, Mahesh M. Mansukhani, Peter L. Nagy, Mariano J. Alvarez, Andrea Califano, Farhad Forouhar, Beata Modzelewski, Chelsey M. Mitchell, Darrell J. Yamashiro, Lianna J. Marks, Julia L. Glade Bender, Andrew L. Kung

**Affiliations:** 1 Department of Pediatrics, Memorial Sloan Kettering Cancer Center, New York, NY 10065, USA; 2 Department of Pediatrics, Columbia University Medical Center, New York, NY 10032 USA; 3 Department of Pathology and Cell Biology, Columbia University Medical Center, New York, NY 10032, USA; 4 Department of Systems Biology, Columbia University Medical Center, New York, NY 10032 USA; 5 Department of Biological Sciences, Columbia University, New York, NY 10027, USA; 6 Darwin Health Inc., New York, NY 10032, USA; 7 Present Address: Medical Neurogenetics Laboratories, Atlanta, GA 30342, USA

**Keywords:** Poorly differentiated carcinoma (PDC), Patient-derived xenograft (PDX) models, Precision medicine, MAX, mTOR, BRAF, Temsirolimus, Whole exome sequencing (WES)

## Abstract

**Background:**

Precision medicine approaches are ideally suited for rare tumors where comprehensive characterization may have diagnostic, prognostic, and therapeutic value. We describe the clinical case and molecular characterization of an adolescent with metastatic poorly differentiated carcinoma (PDC). Given the rarity and poor prognosis associated with PDC in children, we utilized genomic analysis and preclinical models to validate oncogenic drivers and identify molecular vulnerabilities.

**Methods:**

We utilized whole exome sequencing (WES) and transcriptome analysis to identify germline and somatic alterations in the patient’s tumor. In silico and in vitro studies were used to determine the functional consequences of genomic alterations. Primary tumor was used to generate a patient-derived xenograft (PDX) model, which was used for in vivo assessment of predicted therapeutic options.

**Results:**

WES revealed a novel germline frameshift variant (p.E1554fs) in APC, establishing a diagnosis of Gardner syndrome, along with a somatic nonsense (p.R790*) APC mutation in the tumor. Somatic mutations in *TP53*, *MAX*, *BRAF*, *ROS1*, and *RPTOR* were also identified and transcriptome and immunohistochemical analyses suggested hyperactivation of the Wnt/ß-catenin and AKT/mTOR pathways. In silico and biochemical assays demonstrated that the MAX p.R60Q and BRAF p.K483E mutations were activating mutations, whereas the *ROS1* and *RPTOR* mutations were of lower utility for therapeutic targeting. Utilizing a patient-specific PDX model, we demonstrated in vivo activity of mTOR inhibition with temsirolimus and partial response to inhibition of MEK.

**Conclusions:**

This clinical case illustrates the depth of investigation necessary to fully characterize the functional significance of the breadth of alterations identified through genomic analysis.

**Electronic supplementary material:**

The online version of this article (doi:10.1186/s13073-016-0366-0) contains supplementary material, which is available to authorized users.

## Background

Precision medicine approaches are being increasingly utilized in the diagnostic characterization and the development of molecularly informed therapeutic plans in both common and rare cancers [1–9]. Although improved insight into the biology and the refinement of treatment approaches for more commonly encountered cancers are obvious merits of precision medicine, the genomic characterization and the development of individualized treatment plans informed by the mutational status of patients with rare cancers epitomizes the power and potential of precision medicine. To illustrate this view, metastatic carcinomas with an occult primary site of origin represent a diagnostic and therapeutic dilemma for clinicians and are exceedingly rare in children [1, 10]. Undifferentiated or poorly differentiated carcinomas (PDC) are often treated as a single entity using platinum-based combination chemotherapies despite the clinicopathologic heterogeneity of this group of tumors [3]. Attempts have been made to classify PDCs based on immunohistochemical profiles along with clinical presentation in order to assign a putative anatomic site of origin which would then direct site-specific therapy [2]. Several studies have suggested that treatment of cancers of unknown primary site (CUP), which often include PDCs, have improved clinical outcomes when treated with site-specific therapy in comparison to empiric chemotherapy [3, 4].

With advances in molecular diagnostics, the application of next-generation sequencing technologies has enabled deeper insight into the tissue of origin for occult tumors and PDCs as well as offered therapeutic guidance to clinicians. However, despite improvements in the molecular characterization enabled by precision medicine, the biologic significance and the clinical relevance of identified mutations to treatment of the patient often remain unclear without additional investigation. The diagnostic and treatment challenges characteristic of rare tumors, such as CUPs and PDCs, represent a category of disease that would significantly benefit from a precision medicine-based approach to diagnosis and treatment planning. We present a case study of a pediatric PDC that involved the genomic and functional evaluation of identified variants and in vivo assessment of putative targets within the framework of a pediatric precision medicine program.

### Clinical presentation and family history

**Fig. 1:**
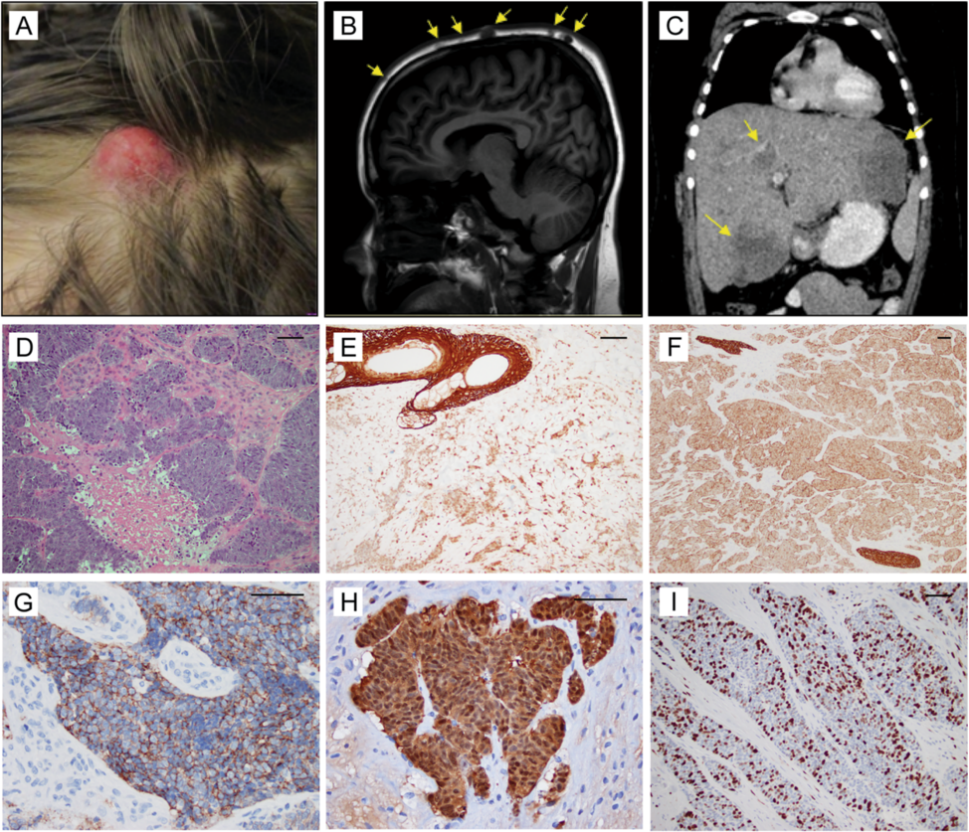
Clinical presentation of metastatic PDC. **a** Representative scalp nodule. **b**, **c** Diagnostic imaging demonstrating the presence of multiple lytic lesions of the calvarium (**b**) as well as heterogeneous lesions within the liver with associated hepatosplenomegaly (**c**). **d**–**i** Immunohistochemical staining consistent with diagnosis of a PDC with high proliferative index: (**d**) H&E (200X), (**e**) cytokeratin 5 (200X), (**f**) cytokeratin 10 (100X), (**g**) EpCAM (400X), (**h**) ß-catenin (400X), (**i**) Ki67 (200X). Scale bar = 100 μm

A 14-year-old boy with a history of asthma, attention deficit-hyperactivity disorder, and recurrent epidermoid cysts of the scalp presented to an outside hospital with a two-month history of malaise and back and abdominal pain which had been increasing in severity. He had also recently developed a progressively enlarging and discolored scalp lesion (Fig. 1a). Initial clinical workup was remarkable for elevated inflammatory markers (elevated erythrocyte sedimentation rate (ESR) and C-reactive protein), hyperuricemia, transaminitis, and elevations in lactate dehydrogenase and gamma-glutamyl transferase (GGT). He had no history of significant weight loss, pruritus, or night sweats, but presented with several days of intermittent fevers for which he had begun empiric antibiotic therapy. A complete blood count showed no abnormalities and levels of carcinoembryonic antigen (CEA), α-fetoprotein (AFP), and ß-human chorionic gonadotropin (ß-HCG) were normal. Diagnostic computed tomography (CT) imaging of the head revealed multi-focal lesions of the scalp, including several discrete, enhancing extra-axial masses and an ill-defined lytic calvarial lesion (Fig. 1b). Magnetic resonance imaging (MRI) also confirmed the multiple scalp lesions as well as multiple vertebral, pelvic, and femoral bone lesions. Due to the patient’s presentation of persistent abdominal pain in the setting of transaminitis and an elevated GGT, an abdominal ultrasound was performed and revealed an enlarged heterogeneous, nodular liver with multiple hypoechoic masses. A subsequent CT scan of the abdomen confirmed the presence of hepatosplenomegaly and infiltrative lesions within the liver (Fig. 1c). A chest X-ray showed no pulmonary lesions. The presenting physical exam was notable for scattered, firm red papules and nodules on the scalp, ranging in size between 5 mm and 3 cm. A distant mobile, subcutaneous nodule (8 mm) was also noted over an elbow. Abdominal exam revealed hepatosplenomegaly. The patient displayed no dysmorphic features and the remainder of the physical exam was unremarkable.

The patient’s past medical history was significant for a history of recurrent epidermoid cysts since the age of 2 years. Over his lifetime, he had over 15 cysts removed with pathology consistent with either an epidermoid cyst or hybrid lesions comprising an epidermoid cyst and pilomatricoma. Review of the family history was notable for multiple members on the maternal side with a history of cancer. The patient’s mother had recurrent breast cancer initially diagnosed at the age of 36 years. Additionally, cases of breast cancer were reported in the maternal grandmother and maternal aunt. A maternal great grandmother was diagnosed with ovarian cancer, a maternal great grandfather and maternal great uncle were diagnosed with lung cancer, a maternal nephew with a “bone cancer,” and a maternal grandfather with colon cancer. Maternal siblings are healthy. The patient’s father was well with no significant medical issues. The paternal family history was significant for a paternal great grandfather and grand uncle diagnosed with lung cancer. The patient had two siblings who were healthy.

A biopsy of the largest scalp lesion localized over the left occiput was performed which revealed the presence of a high-grade carcinoma positive for pancytokeratin (AE1/AE3) and diffuse nuclear staining for ß-catenin with a Ki67 proliferative index of 50 %. The tumor showed focal weak staining for synaptophysin and was negative for chromogranin, CD3, CD20, CD30, CD99, PLAP, P63, myogenin, MART-1, EMA, desmin, CK7, CK20, S100, Oct3/4. INI-1 showed intact nuclear staining. A needle biopsy of the liver lesions was also performed and demonstrated similar histology. Based on the histopathological features, a diagnosis of high-grade carcinoma of unknown origin was rendered.

The patient was transferred to Columbia University Medical Center (CUMC) for further medical management and workup given the rarity and clinical acuity of the diagnosis. Given the relative rarity of high grade carcinoma in children, the patient and family were consented for participation in the Precision In Pediatric Sequencing (PIPseq) program at CUMC. An excisional biopsy of a large scalp lesion was performed to confirm the original diagnosis and excess material was utilized for genomic analysis, as well as for the generation of patient-derived xenograft (PDX) tumor mouse models. Pathologic review of the excisional biopsy scalp lesion demonstrated histologic features similar to the initial biopsy specimen and remained positive for ß-catenin, cytokeratin 5 and 10, and EpCAM (Ber-EP4) with a proliferative index of 50 % (Fig. 1d–i). Additionally, there was focal positivity for synaptophysin and negativity for CK7, CK20, p63, S100, and chromogranin. These findings were considered consistent with a PDC with focal neuroendocrine features.

Based on this histopathological diagnosis, therapy was initiated utilizing a combination of paclitaxel, carboplatin, and etoposide (PCE) administered intravenously and given as 21-day cycles. Stable to partial responses in the scalp, calvarial, vertebral, and hepatic lesions were observed on CT reassessment of all known disease sites after recovery from the first cycle of therapy. Furthermore, the patient reported overall improvement in pain with decreased requirements for opiate therapy. The patient completed ten cycles of PCE therapy with continued disease stabilization. Although all lesions demonstrated overall improvement, the patient remained a partial responder to PCE therapy with persistent lesions in the scalp, calvarium, vertebra, pelvic, femora, and liver. Pain symptoms had largely resolved and the patient was successfully weaned off opiate therapy.

Restaging performed after ten cycles of PCE revealed a new lesion in the brain. Based on genomic analysis of the original tumor, the patient was started on a regimen that incorporated the mTOR (mammalian target of rapamycin) inhibitor, temsirolimus, given in combination with the alkylating agent, temozolomide, and the topoisomerase inhibitor, irinotecan [11]. After a single cycle of therapy, the patient demonstrated radiographic evidence of disease progression and chose to discontinue further therapy. The patient subsequently died of progressive liver failure.

## Methods

### Chemical reagents

Selumetinib, carboplatin, irinotecan, and temsirolimus were purchased from MedChem Express. JQ1 was kindly provided by Dr. James Bradner (Dana-Farber Cancer Institute, Boston, MA, USA). Drugs were resuspended in N-Methyl-2-pyrrolidone (NMP) to create a stock solution (Sigma Aldrich) and diluted in PTD buffer (30 % PEG-400; 5 % Tween 80; 65 % Dextrose water, D5W, Sigma Aldrich) before drug dosing.

### Patient-derived xenograft (PDX) generation

After obtaining a portion of the biopsy specimen, the tumor tissue was fragmented into ~2 mm fragments and implanted subcutaneously into the flanks of NSG-HPRT null mice (NSG-H; NOD.Cg-Prkdc^scid^ Il2rg^tm1Wjl^ Hprt^b-m3^/EshJ, Strain 012480, Jackson Laboratory, Bar Harbor, ME, USA) to generate the passage 0 (P0) generation. When P0 tumors reached a size of ~1 cm in the widest dimension, the PDX tumors were collected after humane euthanasia and expanded into a P1 generation for therapeutic studies (P4 tumors were used for the selumetinib efficacy study). Mice were randomized and assigned to treatment groups once tumors reached a volume of 150–200 mm^3^. Tumor growth was measured biweekly using calipers and mice were euthanized as per institutional animal protocol guidelines at the indicated time points. Tumors were collected and fragments were either fixed in 4 % formalin for histologic analysis or snap frozen in liquid nitrogen for subsequent DNA, RNA, and protein isolation and analyses.

### PDX treatment studies

PDX models were dosed with single agents as follows: carboplatin 16 mg/kg intraperitoneally (IP) twice a week; JQ-1 50 mg/kg IP daily (5 days on/2 days off); temsirolimus 20 mg/kg IP daily; irinotecan 20 mg/kg IP daily (5 days on/2 days off); and selumetinib 50 mg/kg orally (PO) twice daily (5 days on/2 days off). Combination treatment was given with temsirolimus 15 mg/kg IP along with irinotecan 20 mg/kg IP daily (5 days on/2 days off).

### Cell culture

293 T cells (Invitrogen), 293 T platinum E (Cell Biolabs, San Diego, CA, USA), wild-type and BRAF –/– mouse embryonic fibroblasts (MEFs) were maintained in DMEM (Gibco), 10 % FetalPlex™ animal serum complex (Gemini Bio-Products), and 1 % Antibiotic-Antimycotic Solution (Gibco). Wild-type MEF and BRAF –/– MEF were a kind gift of Dr. Catrin Pritchard (University of Leicester, Leicester, UK).

### Immunoblotting

Cells and xenograft tumor samples were resuspended in high SDS-RIPA Buffer (50 mM Tris-HCl, pH 7.5, 150 mM Sodium Chloride, 1 % Triton X-100, 1 % sodium deoxycholate, 1 % SDS, 2 mM EDTA; Sigma Aldrich). Tissues were disrupted and homogenized with a TissueLyser II (Qiagen) for 2 × 2 min intervals at 30Hz. Protein concentration was determined using the Pierce™ BCA Protein Assay Kit (Pierce). A total of 15–50 μg of protein extracts were loaded onto NuPAGE® Novex® 4–12 % Bis-Tris Protein Gels (Life Technologies) and subsequently transferred onto nitrocellulose membranes using the iBlot® Dry Blotting System (Life Technologies). The blots were developed using SuperSignal™ West Pico Chemiluminescent Substrate (Thermo Scientific). Antibodies: S6-Ribosomal protein (5G10), Phospho-S6 Ribosomal protein (Ser240/244) (D68F8), Phospho-4E-BP1 (Thr37/46) (236B4), p44/42 MAPK (Erk1/2) (137 F5), and Phospho-p44/42 MAPK (Erk1/2) (Thr202/Tyr204) (D13.14.4E) were purchased from Cell Signaling Technology. C-MYC (Y69) and N-MYC (NCM II 100) were purchased from Abcam. FLAG (M2) and β-actin (A2066) antibodies were purchased from Sigma Aldrich.

### Immunohistochemistry

Immunohistochemistry was performed by the Columbia University Medical Center Pathology Department and the Herbert Irving Comprehensive Cancer Center Molecular Pathology Core using standard procedures. Antibodies: LC3A/B (D3U4C), cleaved caspase-3 (Asp175), S6-Ribosomal Protein (5G10), and Phospho-S6-Ribosomal Protein (Ser240/244) (D68F8) were purchased from Cell Signaling Technology. Ki-67 (Clone MIB-1) was purchased from Dako. A minimum of five fields per section were analyzed for caspase 3 and Ki67 quantification.

### Transfection and retroviral transduction

pBABEbleo-Flag-BRAF-V600E was kindly provided by Christopher Counter (Addgene, plasmid # 53156). pBabe-bleo-Flag-BRAF-WT and pBABEbleo-FLAG-BRAF-K483E were generated by gene synthesis and cloning (GenScript, Piscataway, NJ, USA). 293 T cells were transfected using Lipofectamine® 3000 (Life Technologies) according to the manufacturer’s instructions.

Retrovirus production and transduction were performed using 293 T platinum E cells following the manufacturer’s instructions and as previously described [12].

### Electrophoretic mobility shift assay (EMSA)

MAX, MAXR60Q, C-MYC, and MXD1 cDNAs were generated by gene synthesis (GenScript) and cloned into pF3A WG (BYDV) Flexi® Vector (Promega). In vitro transcription and translation (IVT) was performed using the TNT® SP6 High-Yield Wheat Germ Protein Expression System (Promega). Wheat germ extracts containing the indicated IVT proteins were incubated in EMSA binding buffer (10 mM Tris-HCl, pH 7.5, 50 mM KCl, 1 mM DTT, 2.5 mM DTT, 0.25 % Tween-20, 50 ng poly(dIdC)), in the presence of 50 ng IRDye-800 labeled probe (Integrated DNA Technologies). Probe sense sequence: 5’-CGGCAGCGAGCCACGTGGACCAACTA-3’. Reactions were loaded onto a 4–12 % TBE gel and imaging was performed on an Odyssey® Fc Imaging System (LI-COR).

### Structural modeling

Visualization and comparison of protein structures and modeling exercises were performed using XtalView. Crystallography and NMR System (CNS) was used for minimization of steric clashes within the heterodimer and between the protein and DNA. All structural figures were made using PyMol [13].

### Nucleic acid extraction, clinical sequencing, and analysis

DNA from macro-dissected paraffin-embedded tumor, OCT-embedded frozen tissue, bone marrow, peripheral whole blood, or buccal swabs was extracted using the QIAGEN QIAamp Tissue Kit (for tissue samples) on the QIAcube system; QIAsymphony DNA Mini Kit (blood and bone marrow); or the QIAGEN DNA Micro Kit (buccal swabs). RNA was extracted using the QIAGEN RNeasy Kit (fresh frozen tissue) or the RNeasy FFPE Kit (paraffin-embedded tissue). All slides were evaluated by a pathologist (AT or MM) to ensure that a minimum of 50 % viable tumor was present for subsequent extraction and analyses. Whole exome sequencing (WES) was performed using the Agilent SureSelectXT All Exon V5 + UTRs capture kit for library generation, and sequenced on the HiSeq 2500 System (Illumina), using paired-end 100 cycle × 2 sequencing. RNA was sequenced using the TruSeq Stranded Total RNA LT Sample Prep Kit (Illumina), with 100 cycles × 2 paired-end sequencing on the HiSeq 2500.

DNA sequencing reads were de-multiplexed and converted to FASTQ files using CASAVA from Illumina. Following mapping and variant calling of both tumor and normal samples by NextGENe, resulting variants were subject to filtering. Variants in normal DNA were passed through a “reference range filter” of cancer predisposition genes, genes relevant to pharmacogenomics, and variants relevant to patient care; a “reportable range filter” which includes COSMIC variants in the patient’s mutation report file and variants in genes on the list of ACMG (American College of Medical Genetics and Genomics) recommendations for reporting of secondary findings; as well as a frequency filter which included variants whose minor allele frequency in The 1000 Genomes [14] is less than 1 %. Somatic mutations in the tumor were identified by subtracting all variants called in normal tissue (output at minor allelic fraction of 5 %) from variants called in the tumor (output at a minor allelic fraction of 10 %). Somatic mutations were further characterized as homozygous, compound heterozygous, “de novo.” or disruptive.

Copy number changes were identified using the EXCAVATOR software 44 [15]. In addition, all high-quality heterozygous variants with allelic ratios of 45–55 % in the normal sample were outputted to allow identification of copy number neutral loss of heterozygosity (LOH) as well as to support the copy number variations (CNV) identified by EXCAVATOR.

### Sanger sequencing

Purified RNA from xenograft tissue samples were reverse-transcribed using ThermoScript™ RT-PCR System for First-Strand cDNA Synthesis (Life Technologies). PCR was performed using Platinum Blue PCR super mix (Life Technologies). Sanger sequencing was performed by Genewiz (South Plainfield, NJ, USA) on PCR products. Primer sequences are available upon request.

### Data interpretation and reporting

Interpretation of clinical WES, RNA sequencing (RNA-seq), and CNV was conducted by a multidisciplinary team representing pediatric oncologists, pathologists, surgeons, molecular and clinical geneticists, and bioinformaticians in the setting of a molecular tumor board.

### Gene expression profile and expression outlier analyses

RNA was prepared using a TruSeq Stranded Total RNA Kit (Illumina). Paired-end sequencing with 100 bp read lengths was performed on an Illumina HiSeq 2500. Transcription level estimation, measured in FPKM (fragments per kilobase per million reads sequenced), was performed by an RNA-seq processing pipeline developed by the Personalized Genomic Medicine Program at CUMC following standard practices. First, reads were bio-informatically filtered for rRNA using a program called SortMeRNA [16] and trimmed to remove poor quality tails using TrimGalore [17]. The remaining reads are then mapped to the human genome (hg19) using the Tuxedo Suite [18], consisting of Bowtie, TopHat, and Cufflinks. Non-uniquely mapped reads are excluded before estimation of FPKM by Cufflinks. For transcriptomic analysis, the Tuxedo Suite Package with custom modifications was used to generate BAM from FASTQ files from CASAVA, and mutation calling performed using NextGENe software. At least 50 million independent mapped reads were required. Transcriptomic variants were used to confirm DNA sequence variants. In addition, unmapped reads were analyzed using “FusionMap” to generate a list of fusions for review by molecular pathologists.

Ranking overexpressed genes was done by an algorithm developed by PGM: DiffExprOutlier. DiffExprOutlier quantitates transcript levels for genes in comparison to the general transcription levels of the tissues examined as determined by 2921 normal RNA-seq samples from the GTEx database (version 4) [19]. For normalization, the median transcription levels (FPKM) of 8000 housekeeping genes are used as a reference [20]. The normalized expression was determined for each gene in each normal sample, as well as in the test sample. For each gene, the test sample was ranked within the normal reference samples based on the relative normalized expression of that gene. Genes that rank the test sample in the top or bottom 10 % of all other samples were outputted for review.

### Publicly available RNA-seq data acquisition and normalization

Messenger RNA (mRNA) expression data (RNA-seq) from 33 tissue types was obtained from The Cancer Genome Atlas (TCGA) [21]. Level-3 raw-counts per gene were obtained from TCGA data portal, normalized to correct for differences in library size and transformed to stabilize the variance by fitting the dispersion to the negative-binomial distribution, as implemented in the DESeq package from Bioconductor [22]. Alternatively, library-size normalized counts per gene were corrected by average transcript size to generate FPKM. RNA-seq data for gastro-entero-pancreatic neuroendocrine tumors were obtained using a HiSeq 2000 sequencer (Illumina). Reads were mapped to the human genome (UCSC-hg19) by Bowtie2 [23, 24] and uniquely mapping reads were summarized at the gene level using the GenomicFeatures package from Bioconductor [25]. Raw-counts per gene were normalized and variance stabilized as described for TCGA data. We performed absolute gene expression discretization by fitting a mixture of two Gaussian models, representing the non-expressed and expressed transcripts, to the probability density of expression, and estimating the relative likelihood of expression from the fitted distributions.

### T-distributed stochastic neighbor embedding (t-SNE)

We used t-SNE [26], as implemented in the t-SNE package from Bioconductor, to generate a two-dimensional (2D) representation of the similarity between samples as measured by correlation analysis in a transformed expression space to highlight the similarity in absolute expression terms. Briefly, the relative likelihood of expression was computed by fitting a mixture of two Gaussian distributions (the first representing very low to non-expressed genes and the second for expressed genes) to the probability density of expression represented as FPKM. This transformation efficiently shrinks the variance between expressed genes while amplifying the variance between expressed and non-expressed genes. In order to reduce computation time, this analysis was performed on 3167 samples, including at most 100 samples per tumor type selected at random from our TCGA pan-cancer expression database and the carcinoid sample under study.

### Statistical analyses

All in vitro experiments were performed at least three times. The statistical significance of differences was determined using the Student’s t-test with a minimal level of significance of *P* < 0.05. Differences in tumor volume response to drug treatments were compared using a two-way ANOVA. Statistical significance of differences in tumor growth among treatment groups was determined by the Mann–Whitney U test using GraphPad Prism 6.0 software. Two-sided *P* values were given at a 95 % significance level.

## Results

### Genomic characterization of primary tumor

**Fig. 2:**
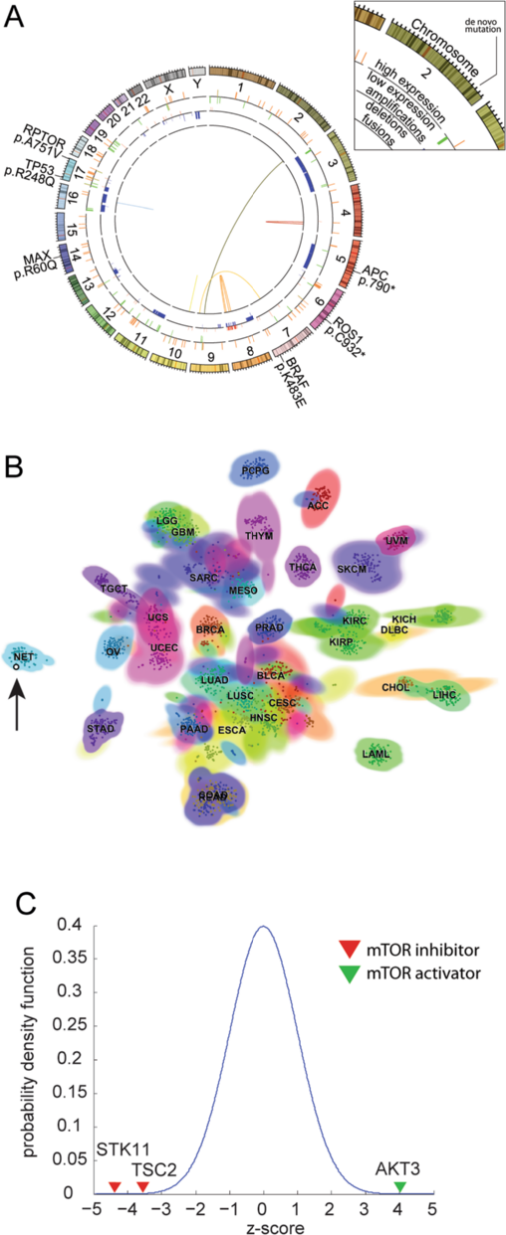
WES and transcriptome sequencing of a primary tumor. **a** *Circos plot* summarizing WES and transcriptome analysis of primary tumor. *Inner circle* represents structural variants and gene fusions; *second tier*, copy number variations (*blue*, loss; *red*, gain); *third tier*, mRNA expression outlier analysis of cancer related genes within the top and bottom 10th percentile (*green*, under-expressed; *orange*, over-expressed); *fourth tier* (*outer circle*), somatic mutations localized to respective chromosomes. **b** *Scatter-plot* showing the t-SNE 2D projection for 3167 samples, including at least 100 samples (indicated in the figure) for each of the 34 tissue types represented in our pan-cancer database. Tissue ID is indicated by different *colors* and the carcinoid sample is indicated by a *bold black dot* and *arrow*. **c** Relative gene expression rank of outlier genes after z-normalization across a compendium of expression profiles from the GTEx database. A z-distribution is superimposed as reference. *ACC* adrenocortical carcinoma, *BLCA* bladder urothelial carcinoma, *BRCA* breast carcinoma, *CESC* cervical carcinoma, *CHOL* cholangiocarcinoma, *COAD* colon adenocarcinoma, *DLBC* diffuse large B-cell lymphoma, *ESCA* esophageal carcinoma, *GBM* glioblastoma multiforme, *HNSC* head and neck carcinoma, *KICH* kidney chromophobe, *KIRC* clear cell carcinoma of the kidney, *KIRP* renal papillary cell carcinoma, *LAML* acute myeloid leukemia, *LGG* low grade glioma, *LIHC* hepatocellular carcinoma, *LUAD* lung adenocarcinoma, *LUSC* lung squamous cell carcinoma, *MESO* mesothelioma, *NET* gastrointestinal neuroendocrine tumor, *OV* ovarian carcinoma, *PAAD* pancreatic adenocarcinoma, *PCPG* pheochromocytoma and paraganglioma, *PRAD* prostate adenocarcinoma, *READ* rectal adenocarcinoma, *SARC* sarcoma, *SKCM* cutaneous melanoma, *STAD* gastric adenocarcinoma, *TGCT* testicular germ cell tumor, *THCA* thyroid carcinoma, *THYM* thymoma, *UCEC* uterine corpus endometrial carcinoma, *UCS* uterine carcinosarcoma, *UVM* uveal melanoma

Primary tumor tissue obtained from a scalp biopsy was processed for routine histopathological diagnostic evaluation, genomic analysis, and generation of a PDX model. Genomic analysis comprised tumor/normal WES and RNA sequencing of the tumor. Variant calls were independently determined for tumor and germline, and somatic variants determined based on subtraction. WES data were used to determine CNV and RNA-seq was mined to identify translocations and gene expression outliers by comparison to an expression model derived from the genotype-tissue expression database (GTEx) [27]. Genomic alterations identified through this analysis are summarized in Fig. 2a. Datasets are available through the cBioPortal for Cancer Genomics (http://cbioportal.org) [28, 29].

#### Germline variants and somatic alterations

A frameshift variant in *APC* (c.4660_4661insA, p.E1554fs) was identified in both the normal and tumor material and was determined to be a de novo germline mutation after sequencing of both parents. This finding supports a diagnosis of familial adenomatous polyposis (FAP)/Gardner syndrome. A second mutation in the APC tumor suppressor was identified (c.2368A > T, p.R790*) in the tumor. Additional somatic mutations in cancer-associated genes included missense mutations in *TP53* (c.743G > A, p.R248Q), *MAX* (c.179G > A, p.R60Q), *BRAF* (c.1447A > G, p.K483E), and *RPTOR* (c.2252C > T, p.A751V), and a nonsense mutation in *ROS1* (c.1176 T > A, p.C392*). The *TP53* (p.R248Q) and APC (p.R790*) mutations had allelic frequencies consistent with loss of heterozygosity (LOH).

The identified *TP53* (p.R248Q) mutation is a previously described gain-of-function mutation which is associated with early-onset development of many tumor types [30–32]. The somatic *APC* (p.R790*) mutation has also been previously reported in the Catalogue Of Somatic Mutations In Cancer (COSMIC) database [33, 34]. The newly identified de novo germline *APC* (p.E1554fs) mutation is localized on a codon where other frameshift mutations have been reported in COSMIC. Both *APC* mutations generate truncated proteins resulting in constitutive activation of canonical WNT pathway signaling. Immunohistochemical analysis of primary tumor showed diffuse ß-catenin nuclear staining (Fig. 1h) consistent with the described genetic lesions.

Given the role of MET in the progression of CUPs, we also evaluated the status of MET in the primary tumor [35, 36]. Analysis of *MET* revealed no evidence of amplification or other gene alterations (data not shown).

#### Copy number variation

Several segmental changes consistent with chromosomal instability were identified including -3, -5q, 8q, del(9p), -11p, del(11q), del(13q), -16,-17p, del(21q), and -Y. Among the genes localized within deleted regions are well-established tumor suppressor genes including the cell cycle inhibitors *CDKN2A* and *RB1* and the mTOR inhibitor *TSC2*. Consistent with LOH suggested by the high allelic frequencies for *TP53* (p.R248Q) and *APC* (p.R790*) mutations, we confirmed segmental loss of -17p and -5q containing the wild-type *TP53* and *APC* (p.E1554fs) alleles, respectively. Finally, we observed a copy gain in the 8q region containing the *MYC* locus.

#### Gene expression analysis

To better understand the tissue of origin for the tumor, we used clustering to map the gene expression profile of the patient’s tumor to all tumor samples available in the TCGA dataset and an additional cohort of neuroendocrine tumors (NET). The patient’s transcriptome showed the highest similarity to a cohort of 212 enteropancreatic NETs representing primary tumors and liver metastases originating from pancreatic, small intestine, and colorectal primaries (Fig. 2b), providing support for the classification of this tumor as a PDC with neuroendocrine features.

To identify gene expression outliers (over-expressed and under-expressed), we compared the gene expression profile from the tumor with a model constructed from the mean expression of all genes derived from the GTEx database of 2921 transcriptomes. Gene expression outlier analysis showed several genes involved in the mTOR pathway which ranked in the top and lower 10th percentiles (Fig 2c). Notably, we observed over-expression of *AKT3* (an mTOR activator) and reduced expression of *TSC2* (consistent with single copy loss of the gene) and *STK11* which both inhibit mTOR activity (Fig. 2c). These results suggest hyperactivation of the mTOR pathway, which was verified by immunohistochemical evaluation of the primary tumor demonstrating elevated phosphorylation levels of ribosomal protein S6 (RPS6), a biomarker of mTOR activation (Additional file 1: Figure S1). No other aberrant expression of genes involved in CUP progression, such as *MET*, was observed.

#### Protein fusion events

Fusion events were observed but did not involve known cancer related genes or targetable signaling pathways. Most fusion events were intra-chromosomal and occurred within the region of chromosome 8q (Fig. 2a).

#### Functional characterization of MAX p.R60Q

**Fig. 3:**
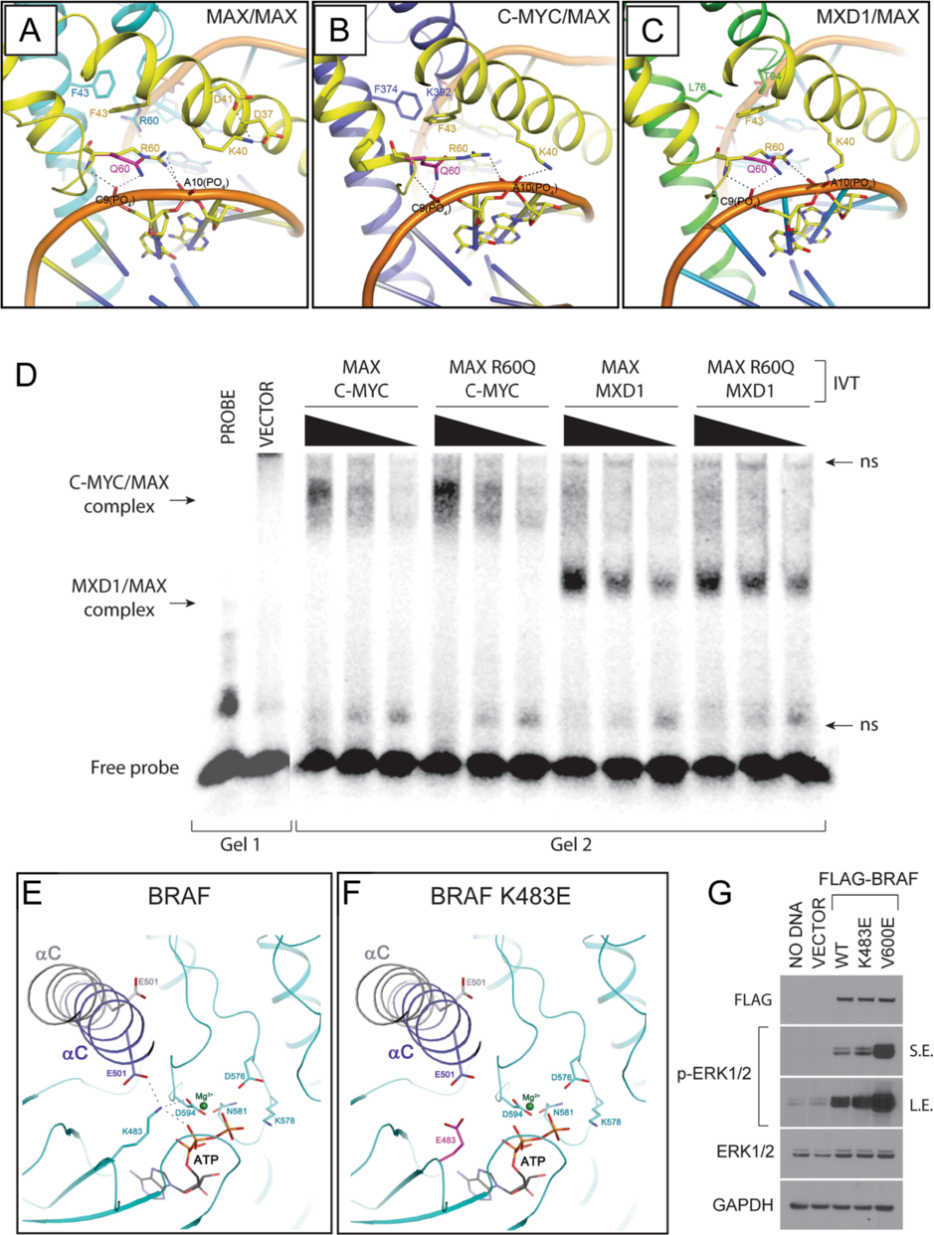
Structural and functional analyses of Variants of Unknown Significance (VUS). **a**–**c** Structures of MAX homodimer and C-MYC-MAX and MXD1-MAX heterodimers in complex with DNA. **a** MAX-MAX homodimer crystal structure (PDB id: 1AN2) in which the subunit A (*yellow* for carbon atoms) and B (*cyan* for carbon atoms) are represented and the side chains of several invariant residues are depicted with stick models and labeled. **b** Crystal structure of C-MYC/-MAX heterodimer in complex with DNA (PDB id: 1NKP). MAX and C-MYC carbon atoms are represented in *yellow* and *purple*, respectively. **c** Crystal structure of MAX-MXD1 heterodimer in complex with DNA (PDB id: 1NLW). MAX and MXD1 carbon atoms are represented in *yellow* and *green*, respectively. In all structures presented, the MAX p.R60Q mutation is shown in *magenta. Dashed lines* (*black* and *magenta*) represent hydrogen bonds. The sugar-phosphate backbone of DNA is shown in *orange* with two selected nucleotides from each subunit shown as stick models. **d** MAX^R60Q^ mutant heterodimerizes with C-MYC and MXD1 and binds to DNA. The indicated proteins were transcribed and translated in vitro and incubated with an E-box containing probe. Specific proteins/DNA complex bands are indicated on the *left*. Non-specific (ns) binding products present in the probe-only and vector control lanes are indicated on the *left*. **e**, **f** Structures of wild-type BRAF and BRAF p.K483E mutant. **e** Model of the BRAF kinase domain in complex with ATP (*black* for carbon atoms) and a Mg^2+^ ion (*dark green*), in which the side chains of five essential residues in BRAF, are shown, and labeled. The helix αC in its active conformation (*dark violet*) (PDB id: 4MNE) and in inactive conformation (*light gray*) (PDB id: 4WO5) is represented as cartoon and the side chain of the invariant E501 is depicted with stick models in two orientations. **f** Model of the BRAF kinase domain in which K483 is replaced by E (*magenta* for carbon atoms). **g** Proteins levels and phosphorylation level of ERK1/2 upon transient transfection of the indicated BRAF proteins in HEK 293 T cells

MAX is a transcription factor of the basic helix-loop-helix leucine zipper (bHLH-LZ) family which is the obligate heterodimer for C-MYC, N-MYC, and L-MYC oncoproteins. MAX can also form a homodimer or heterodimerize with MXDs and MNT which functionally antagonize MYC/MAX activity and promotes cell cycle arrest and differentiation (reviewed in [37, 38]). *MAX* (p.R60Q) is the most common *MAX* gene mutation reported in the COSMIC database suggesting it may promote MAX oncogenic activity [39]. Arg 60 is the first amino acid of Helix 2 and participates in crucial protein–protein and protein–DNA interactions necessary for both homodimerization and DNA binding [40]. To gain insight into the functional consequence of the *MAX* (p.R60Q) mutation, we performed in silico modeling of the mutation in the context of either the MAX homodimer or MAX/C-MYC and MAX/MXD1 heterodimers using published crystal structures [41] (Fig. 3a–c). The structure of the MAX homodimer in complex with DNA [40] confirms that the Arg 60 of each subunit plays a critical role in the stability of the DNA-bound MAX-MAX homodimer complex. Arg 60 forms two hydrogen bonds (H-bond) with the phosphate moiety of DNA in addition to forming π-π bond interactions with the invariant Phe 43 in each subunit (Fig. 3a). Therefore, the mutation of two invariant Arg 60 residues to glutamine at the MAX homodimer–DNA interface disrupts the continuity of the π-π interactions and inhibits both homodimerization and DNA binding. Consistent with our analysis, mutation of the equivalent basic amino acid at the beginning of Helix 2 in the bHLH transcription factor TCF3 abolishes its ability to dimerize and bind DNA [42]. Additionally, overexpression of the MAX^R60Q^ mutant in pheochromocytoma PC12 cells, which lack endogenous wild-type MAX, is unable to repress the expression of an E-box-dependent luciferase reporter [43].

While the MAX p.R60Q mutation abolishes MAX homodimerization and DNA binding, its effects on MAX heterodimerization with C-MYC or other MYC family members is unknown. Structural comparison of DNA-bound MAX/MAX, C-MYC/MAX, or MXD1/MAX reveals significant differences in the dimer interfaces which correspond to the substitution of Arg 60 and Ala 61 of MAX with Lys 392 and Val 393 in C-MYC (Fig. 3b) and Thr 94 and Leu 95 in MXD1 (Fig. 3c), respectively. Lys 392 of C-MYC forms a strong H-bond with the phosphate group of DNA and its long hydrophobic side chain interacts with Phe 374 within C-MYC itself. In addition, the structure of the C-MYC/MAX heterodimer shows that Val 393 of C-MYC forms stronger hydrophobic interactions with Ile 39 of MAX as compared with the hydrophobic interaction between Ile 39 from subunit A of MAX with Ala 61 of subunit B of MAX in the MAX homodimer. Similar interactions are established by MXD1 Thr 94 and Leu 95 within the MXD1/MAX heterodimer. Taken together, these compensated interactions reinforce the heterodimeric assembly in the C-MYC/MAX and MXD1/MAX heterodimers.

In addition, while MAX Lys 40 does not interact with DNA in either subunit of the MAX homodimer (Fig. 3a), our model shows that MAX Lys 40, in the context of C-MYC/MAX and MXD1/MAX heterodimers, forms H-bonds with DNA (Fig. 3b, c) compensating the loss of the H-bond between Arg 60 and DNA in the MAX p.R60Q mutant. Overall, our analysis predicts that the MAX p.R60Q mutant could form a stable heterodimer with both C-MYC and MXD1. To formally evaluate if MAX p.R60Q could form functional heterodimers with C-MYC or MXD proteins, we expressed in vitro either C-MYC or MXD1 proteins in the presence of wild-type MAX or the mutant MAX^R60Q^ (Additional file 1: Figure S2A). Evaluation by EMSA demonstrates that both MAX and MAX^R60Q^ can equally dimerize with C-MYC and MXD1 and bind DNA (Fig. 3d). Hence, these structural and biochemical results suggest that the MAX p.R60Q mutation inhibits MAX homodimerization, but does not disrupt C-MYC/MAX heterodimerization, shifting the balance towards C-MYC activation in proliferating cells.

#### Structural assessment of RPTOR p.A751V

RPTOR, mTOR, and MLST8 constitute the core subunits of the mammalian TORC1 (mTORC1) complex which play a major role in the control of cell growth and metabolism and is often deregulated in cancer [44–46]. RPTOR is a critical component of the mTOR complex and regulates the catalytic activity and substrate recognition of mTOR [47, 48]. Analysis of the identified RPTOR p.A751V mutation by PolyPhen, SIFT, and PROVEAN [49–51] indicates that this mutation is tolerable and not likely to disrupt protein structure/function. This prediction is tenable given the similar sizes, hydrophobicity, and ionization status of alanine and valine. Furthermore, the RPTOR p.A751V residue is localized to a flexible polypeptide stretch that connects RPTOR’s armadillo and β-propeller domains and is contained within a region that does not directly contact either mTOR or mTOR substrates [52]. In addition, the RPTOR p.A751V mutation has been reported in The 1000 Genomes project database indicating that RPTOR p.A751V may represent a genetic variant of RPTOR that is present within the human population [53]. Hence, RPTOR p.A751V mutation would not be expected to negatively affect mTOR pathway activity and contribute to aberrant mTOR signaling.

#### Functional characterization of BRAF p.K483E

BRAF is one of the most frequently mutated genes in cancer [54, 55]. Mutations affecting BRAF normally result in aberrant activation of the downstream MEK/ERK pathway [56]. The identified BRAF p.K483E mutation in the patient’s tumor is localized to the BRAF kinase domain and is predicted to be deleterious by Polyphen, SIFT, and PROVEAN. Modeling of BRAF in complex with ATP (Fig. 3e and Additional file 1: Figure S2B) shows that Lys 483 makes several H-bonds critical for the proper orientation of the ATP molecule within the hydrophobic pocket. When Lys 483 is mutated to glutamic acid (Fig. 3f), the H-bonding network between Lys 483 and the surrounding residues is abolished which has a detrimental effect on the kinase activity. Since both BRAF p.K483M and BRAF p.D594A mutants (Additional file 1: Figure S2C, D) disrupt ATP binding and have been shown to be catalytically inactive [57, 58], we predicted that the BRAF p.K483E mutation is a kinase inactivating mutation. However, given that the BRAF p.K483E mutation is present at the high allelic frequency in the patient’s tumor (>40 %), it has previously been described in chronic lymphocytic leukemia and is catalogued in the COSMIC database suggests that BRAF p.K483E may paradoxically result in downstream pathway activation [59].

To functionally evaluate the consequence of the BRAF p.K483E mutation, we transiently expressed wild-type BRAF, mutant BRAF^K483E^, and the constitutively active BRAF^V600E^ mutant in 293 T cells and evaluated ERK1/2 activation. Compared to a control vector, BRAF^K483E^ expression increased the phosphorylation levels of ERK1/2 although at levels comparable to those induced by wild-type BRAF (Fig. 3g). We also confirmed that the BRAF^K483E^ mutant is able to activate ERK1/2 signaling in wild-type MEFs as well as BRAF null MEFs (Additional file 1: Figure S2E). Since the BRAF^K483E^ mutant is catalytically dead, ERK1/2 activation likely results from allosteric activation of wild-type BRAF or CRAF given the increased ERK1/2 phosphorylation observed in BRAF null cells. These results suggest that BRAF p.K483E mutation activates downstream signaling through the MEK/ERK pathway.

### Preclinical assessment of therapeutic targeting of mTOR, MEK, and MYC pathways

We generated a PDX model and used Sanger sequencing to verify the presence of mutations present in the original tumor (Additional file 1: Figure S3). In light of the genomic profiling studies suggesting alteration of mTOR, BRAF, and C-MYC activity, we evaluated targeted inhibition of these pathways in the PDX model. We decided to test the mTOR inhibitor temsirolimus, the BET inhibitor JQ1, which has been shown to have efficacy on tumors with deregulated C-MYC and N-MYC activity, and the MEK inhibitor selumetinib which inhibits the activity of the MEK/ERK pathway downstream of BRAF [60].

**Fig. 4:**
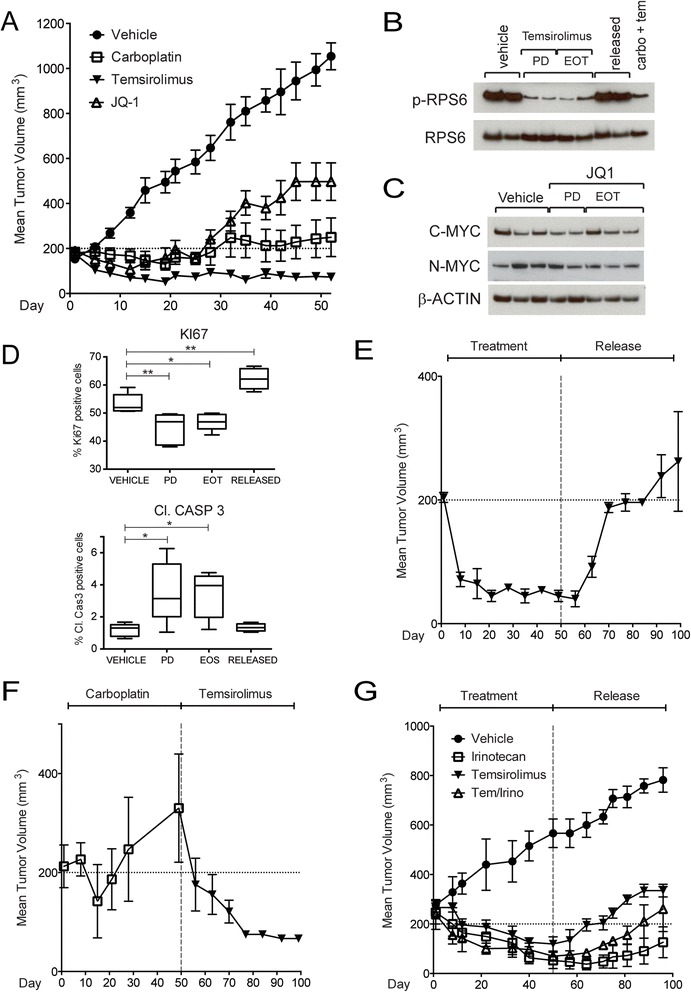
**a** Sensitivity of PDX tumors to the mTOR inhibitor, temsirolimus. Chemoresistance to carboplatin and JQ1 were observed following a transient period of response. Mean and standard error of the mean (SEM) are shown. **b** Phosphorylation level of RPS6 upon temsirolimus treatment. **c** C-MYC and N-MYC protein levels upon JQ1 treatment. **d** Temsirolimus treatment results in decreased Ki-67 staining with concomitant increase in cleaved caspase 3 (Cl. CASP 3) following short-term (3 days) and long-term (50 days) treatments. * *p* < 0.05, ** *p* < 0.01. **e** Tumor growth after temsirolimus treatment withdrawal. Mean and SEM are shown. **f** Temsirolimus treatment can successfully rescue and induce tumor regression in carboplatin-resistant tumors. Mean and SEM are shown. **g** Combination therapy (temsirolimus and irinotecan) does not result in increased anti-tumor activity. Tumor regrowth is observed with withdrawal of treatment. Mean and SEM are shown

PDX tumors exhibited differential sensitivities to treatment with selected inhibitors. In contrast to tumors treated with either vehicle or a standard-of-care chemotherapy agent, carboplatin, treatment with temsirolimus consistently demonstrated abrogation of tumor growth (Fig. 4a). Interestingly, the anti-proliferative effect of temsirolimus was sustained in comparison to carboplatin-treated and JQ1-treated tumors which showed an initial phase of anti-tumor response followed by the emergence of resistance despite continued treatment. Animals treated with the MEK inhibitor selumetinib showed a modest anti-tumor effect but whose overall treatment response would be deemed progressive disease (Additional file 1: Figure S4A). We confirmed effective target engagement in temsirolimus-treated tumors by showing reduced phosphorylation of downstream mTOR targets, RPS6 and 4EBP1, and an associated increase in autophagy (LC3A/B) (Fig. 4b and Additional file 1: Figure S4B). We also show a reduction in activated ERK1/2 (p-ERK1/2) in selumetinib-treated tumors suggesting that inhibition of ERK1/2 signaling is not sufficient to completely abrogate tumor growth in this model (Additional file 1: Figure S4C). JQ1 treatment did not reduce either C-MYC or N-MYC expression (Fig. 4c), indicating that the anti-proliferative effect of JQ1 on tumor growth may not be directly related to modulating MYC expression.

Analysis of the tumor proliferative index in the temsirolimus-treated tumor samples confirmed a decrease in proliferative rate, as indicated by reduced Ki67 staining, with an associated increase in apoptosis (increased cleaved caspase 3) in both short-term and long-term treated tumors (Fig. 4d). However, the anti-proliferative effect of temsirolimus is contingent on continued treatment and suppression of mTOR pathway signaling. We observed that upon withdrawal of temsirolimus treatment, there was a resumption of tumor growth in the PDX model (Fig. 4e) with consequent increase in proliferative activity (Fig. 4d), decrease in apoptosis (Fig. 4d), and re-activation of mTOR pathway signaling (Fig. 4b and Additional file 1: Figure S4B). The overall response to temsirolimus treatment is best characterized as a partial response, with residual tumor remaining despite ongoing therapy (Fig. 4a).

### Modeling chemoresistance and evaluating retrieval therapy

The lack of durable clinical responses in patients with PDC treated with conventional chemotherapy regimens, such as PCE, underscores the need for identifying effective salvage therapies. Hence, models of chemoresistant tumors serve as valuable resources for testing salvage therapy approaches. We used the PDX model to determine if temsirolimus would be efficacious in relapsed disease after conventional chemotherapy. After a treatment period of ~30 days, carboplatin-treated tumors developed resistance manifested by a resurgence of tumor growth despite continued treatment with carboplatin (Fig. 4a). When carboplatin-resistant animals were crossed over to treatment with temsirolimus, tumor regression was evident demonstrating a lack of cross-resistance between the two drugs (Fig. 4f). However, similar to the effects of single agent temsirolimus treatment on naive PDX tumors, the anti-proliferative effects and inhibition of mTOR pathway proteins by temsirolimus remained effective only with sustained exposure to drug (data not shown).

When the patient progressed on PCE therapy, he was changed to a multiagent regimen that included a topoisomerase I inhibitor (irinotecan) in combination with mTOR inhibition which has been used for the treatment of various solid tumors [11, 61–64] and based on findings from our genomic and preclinical studies. The patient rapidly progressed, suggesting that the combinatorial strategy may not have produced the desired additive effect. Therefore, we evaluated the combination of temsirolimus and irinotecan in our PDX model. Tumors treated with the combination of irinotecan and temsirolimus showed no additional anti-tumor effect compared to either agent alone (Fig. 4g). Following withdrawal of treatment, the rates of tumor regrowth were similar between single agent and combination-treated tumors (Fig. 4g). Hence, despite the single agent activity of temsirolimus and irinotecan in these tumors, combined treatment with both agents produced a non-additive effect. In fact, the rate of regrowth after cessation of therapy was faster than irinotecan monotherapy, suggesting an antagonistic effect in the combination. These results paralleled the lack of clinical response observed in the source patient who received treatment with a combination containing the combination of irinotecan and temsirolimus in addition to the alkylating agent, temozolomide.

## Discussion

The rarity of carcinomas in children has made it challenging to determine effective treatments for this group of cancers. Furthermore, the lack of a primary site of disease often complicates the determination of a diagnosis and development of a treatment plan. A review of pediatric cancers treated at a single institution found that only ~0.2 % of cases would be categorized as an undifferentiated or PDC [10]. Hence, the literature provides very little guidance regarding the appropriate treatment of undifferentiated/PDCs in children. The adult experience for PDCs with unknown primary site does offer some insight into the management and treatment of these diseases [3, 4, 65, 66]. Platinum-based combination chemotherapies have generally been used to treat undifferentiated CUPs with modest response rates of 25–35 % and survival outcomes in the range of 6–16 months [67–69]. However, with the development and refinement of next-generation sequencing technologies, there has been a movement towards the genetic characterization of undifferentiated or PDCs with the hope of identifying driver mutations that would inform treatment recommendations [1, 3, 65]. Faced with a general lack of preclinical and clinical information for treating PDCs in a child, we adopted a precision medicine approach to molecularly profile and functionally characterize identified variants in the tumor of the adolescent presented in this report.

We identified lesions involving the mTOR, MEK/ERK, and MYC signaling pathways. Interrogation of identified somatic mutations in *MAX* (p.R60Q) and *BRAF* (p.K483E) predicted these mutations to be deleterious based on computational predictive tools such as PROVEAN, SIFT, and PolyPhen. However, determining the functional consequences of identified mutations or VUSs require further molecular and biochemical investigation. In the case of the *MAX* (p.R60Q) and *BRAF* (p.K483E) mutations, in silico modeling of the mutations in conjunction with biochemical assays suggest that these mutations are likely activating their associated pathways.

*MAX* has recently been identified as a new susceptibility gene in hereditary pheochromocytoma (PCC) [70]. De novo mutations in *MAX* have also been implicated in sporadic PCC [71]. MAX is the central hub of the MYC-MAX-MXD1 network. Within this network, MAX homodimers repress the expression of C-MYC target genes through competition with C-MYC-MAX heterodimers for DNA binding [72, 73]. Our analysis confirms previous studies showing that the MAX p.R60Q mutation disrupts the ability of MAX to homodimerize [43], and we further show that MAX p.R60Q retains the ability to efficiently bind C-MYC. Hence, the MAX p.R60Q mutation promotes an imbalance of the MAX transcriptional network by reducing the intracellular concentration of repressive MAX homodimers without affecting the ability to heterodimerize with C-MYC.

Mutant BRAF proteins normally function as either activated monomers (e.g. BRAF p.V600E) or constitutive dimers with wild-type BRAF and CRAF [57, 74, 75]. In the latter case, even BRAF mutants with no kinase activity, such as BRAF p.D594A, are able to promote ERK phosphorylation by favoring the activation of the other protomer of the dimer [58, 76]. We demonstrated that BRAF p.K483E expression increased ERK1/2 activation despite the BRAF p.K483E mutant harboring a catalytically dead kinase domain. Therefore, ERK1/2 activation may result from allosteric activation of wild-type BRAF or CRAF. This finding is consistent with previous reports showing that BRAF mutants with reduced or no kinase activity are weak activators of ERK1/2 signaling [57, 76]. Additionally, a paradoxical activation of ERK1/2 has also been observed in wild-type BRAF tumors treated with BRAF inhibitors [77]. Therefore, we conclude that BRAF p.K483E is an activating mutation with effects likely mediated through allosteric activation of its dimer partner.

Two mutations in APC, including a somatic nonsense mutation (p.R790*) as well as a novel germline frameshift variant (p.E1554fs), were identified supporting a diagnosis of Gardner syndrome. Despite a strong family history of cancer in the index patient, the APC germline mutation was determined to be a de novo event after constitutional sequencing of the patient’s parents. The novel germline frameshift mutation is localized to a codon where other previously reported frameshift mutations have been observed and catalogued in COSMIC.

In addition to in silico, biochemical, and cell biological analyses, PDX tumor models represent an investigative tool that can be used to test biological and therapeutic hypotheses. We used the patient’s PDX model to assess the utility of JQ1 (a small molecule bromodomain inhibitor) and selumetinib (MEK inhibitor) as potential therapies. Although there were initial responses to both JQ1 and selumetinib, the magnitude and durability of effect were modest and insufficient, as single agents, to obtain a durable response. In contrast, treatment of PDXs with the mTOR inhibitor, temsirolimus, induced a durable partial response. Notably, PDCs with neuroendocrine features and gastrointestinal PDCs have shown hyperactivation of the AKT/mTOR pathway [78–82]. Additionally, given the role of *MET* in the progression of CUPs and the availability of MET-inhibitors in the clinic [35, 36], we evaluated *MET* status in both the primary patient tumor and PDX tumor models, but found no evidence of genetic or expression abnormalities.

When the patient progressed on standard therapy, he was switched to a temsirolimus-containing combination (Tem/TMZ/Irino) which has shown efficacy in various pediatric solid tumors including sustained responses in neuroblastoma, Ewing sarcoma, and ependymoma [11]. However, the patient’s tumor progressed on triple combination therapy leading us to evaluate the combination of temsirolimus and irinotecan in the patient PDX model. Our preclinical studies showed that combination treatment provided no additional anti-tumor effect than either single agent alone, suggesting an antagonistic interaction between temsirolimus and irinotecan. An antagonistic interaction between temsirolimus and irinotecan has also been observed in carcinoma models [83]. These results suggest that preclinical PDX models should play a role in a precision medicine paradigm for evaluating the in vivo efficacy of drugs in clinically relevant combinations as a complement to the evaluation of individual drugs.

## Conclusions

Advances in genome-scale sequencing now allow for the identification of key molecular alterations for patients with cancer. However, the existing methods for inferring functional consequences of genomic alterations is insufficient and many variants in cancer-associated genes are relegated as VUSs. The systematic evaluation of VUSs using structural, in silico, in vitro and in vivo assays is paramount to fully define the functional significance of genomic alterations. Furthermore, the development of PDX tumor models, which have demonstrated correlation between drug activity in the PDX model and clinical outcome [17, 84], is an investigational tool that can be used to evaluate therapeutic hypotheses that emanate from the genomic and functional analyses. This clinical case illustrates the challenges of translating the genomic profile for any given patient into clinical recommendations. The functional validation of VUSs, in vitro assessment of potential therapeutic approaches, and finally in vivo experimental therapeutic studies necessitates months of resource-intensive studies. Moreover, a reductionist experimental approach does not adequately model the complex reality of treating patients in the clinic, necessitating incorporation of approaches to identify synergistic combinatorial therapies. While the timeline for completion of preclinical validation studies may not match the clinical needs of the individual patient, the knowledge gained will be immediately applicable to future patients by converting an increasing number of variants of unknown significance to variants of known significance.

## Additional file

Additional file 1: Figure S1. Activation of mTOR pathway in patient tumor sample. **Figure S2 A.** Levels of in vitro translated MAX, MAX^R60Q^, C-MYC, and MXD1 in samples used for EMSA. **B** Modeling BRAF kinase domain complexed with ATP and Mg^2+^. **Figure S3.** Validation of PDX tumor model by Sanger sequencing. **Figure S4.** Tumor response to selumetinib. (PDF 7254 kb)

## Abbreviations

ACMG: American College of Medical Genetics
AFP: Alpha fetoprotein
bHLH: Basic helix-loop-helix
bHLHLZ: Basic helix-loop–helix leucine zipper domain
CEA: Carcinoembryonic antigen
CNV: Copy number variations
COSMIC: Catalogue Of Somatic Mutations In Cancer
CT: Computed tomography
CUMC: Columbia University Medical Center
CUP: Cancers of unknown primary site
EMSA: Electrophoretic mobility shift assay
FAP: Familial adenomatous polyposis
FPKM: Fragments per kilobase per million reads sequenced
GGT: Gamma-glutamyl transferase
IACUC: Institutional Animal Care and Use Committee
IP: Intraperitoneally
Irino: Irinotecan
LOH: Loss of heterozygosity
mTOR: Mammalian target of rapamycin
NET: Neuroendocrine tumor
NMP: N-Methyl-2-pyrrolidone
NSG: Non-obese severe combined immunodeficiency gamma null mouse
NSG-H: NSG hypoxanthine phosphoribosyl transferase null mouse
P0: Passage 0 generation
PCC: Pheochromocytoma
PCE: Paclitaxel, Carboplatin, Etoposide
PDC: Poorly differentiated carcinoma
PDX: Patient-derived xenograft
PGM: Personalized Genomic Medicine program
PIPseq: Precision in Pediatric Sequencing
PO: Per os/Orally
PTD buffer: PEG-400, Tween 80, Dextrose water
RPS6: Ribosomal protein S6
ß-HCG: beta-human chorionic gonadotropin
TCGA: The Cancer Genome Atlas
Tem: Temsirolimus
Tem/TMZ/Irino: Temsirolimus, temozolomide, irinotecan
TMZ: Temozolomide
t-SNE: T-Distributed stochastic neighbor embedding
VUS: Variants of unknown significance
WES: Whole exome sequencing
