# Supplementary material for: A case study of an integrative genomic and experimental therapeutic approach for rare tumors: identification of vulnerabilities in a pediatric poorly differentiated carcinoma
Source: Genome Med. 2016 Oct 31;8:116. doi: 10.1186/s13073-016-0366-0 (PMC5088685; doi:10.1186/s13073-016-0366-0)
Supplement: Additional file 1: Figure S1. — Activation of mTOR pathway in patient tumor sample. Figure S2 A. Levels of in vitro translated MAX, MAXR60Q, C-MYC, and MXD1 in samples used for EMSA. B Modeling BRAF kinase domain complexed with ATP and Mg2+. Figure S3. Validation of PDX tumor model by Sanger sequencing. Figure S4. Tumor response to selumetinib. (PDF 7254 kb) [file 13073_2016_366_MOESM1_ESM.pdf]

Supplementary Fig 1

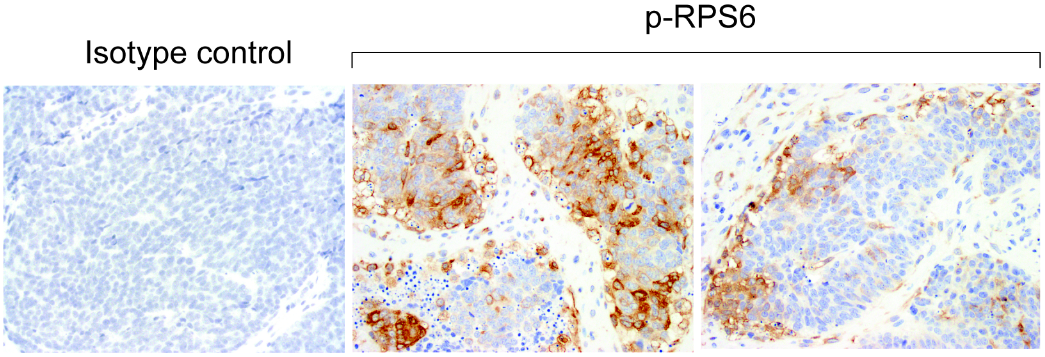


**Supplementary Fig. 1: Activation of mTOR pathway in patient tumor sample.**

Phospho-Ribosomal Protein S6 (P-RPS6). Isotype control (purified rabbit IgG).

Supplementary figure 2

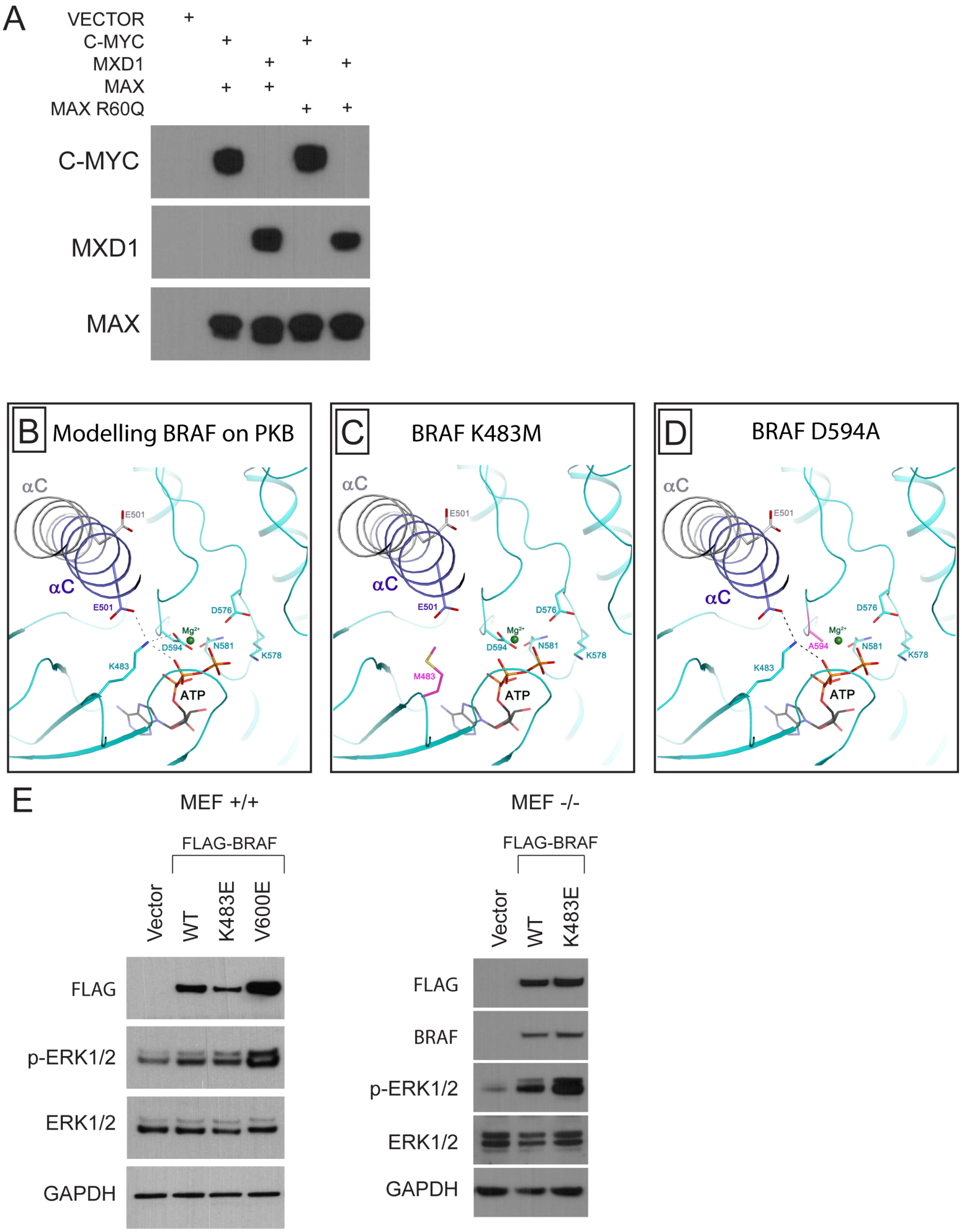

**Supplementary Figure 2: (A)** Levels of *in vitro* translated MAX, $MAX^{R60Q}$, C-MYC and MXD1 in samples used for EMSA. **(B)** Modelling BRAF kinase domain complexed with ATP and $Mg^{+}$. There are currently no available structures of the BRAF kinase domain in complex with ATP and $Mg^{2+}$ or an ATP analog with a metal ion. Therefore, crystal structures of the kinase domain from the protein kinase AKT/PKB (PDB id: 1O6K) [1], tyrosine protein kinase (PDB id: 2SRC) [2], and EGFR kinase (PDB id: 2GS7) [3] were evaluated to provide insight into the mode of ATP-$Mg^{2+}$ binding in the BRAF kinase domain. Comparison of crystal structures using the DALI server [4] revealed that these proteins are close structural homologs of the BRAF kinase domain. The structure of PKB in complex with an ATP analog and two metal ions provides the closest structural model to the BRAF kinase domain complexed with ATP and $Mg^{2+}$. Specifically, the PKB kinase domain (PDB id: 1O6K) in the active conformation is bound to an ATP analog (AMP-PNP) and two metal ions ($Mn^{2+}$) along with six invariant residues (K181, E200, D275, K277, N280, and D293) in its active site which corresponds with six equivalent residues (K483, E501, D576, K578, N581, and D594) in the BRAF kinase domain. The structure of the MEK-BRAF complex (PDB id: 4MNE) [5] provides the most complete structure of the BRAF kinase domain to date. Therefore, data from the MEK-BRAF complex was used as a template for building a complete model of the BRAF kinase domain and guided the construction and assignment of the missing residues (S465-F468) of the P-loop for BRAF. The structure of PKB (PDB id: 1O6K) was also used as a guide for modeling the conformation of the P-loop and positions of ATP and $Mg^{2+}$ in BRAF using XtalView [6], and refined by CNS [7]. Evaluation of constructed crystal structure models suggests that the phosphate moiety of ATP may adopt a *cis* conformation as commonly seen in kinase structures. **(C)** Model of BRAF p.K483M mutant. In comparison to wild-type BRAF, the BRAF p.K483M mutant is characterized by the loss of a critical H-bond resulting in loss of ATP binding. **(D)** Model of BRAF p.D594A mutant. In comparison to

wild-type BRAF, the BRAF p.D594A mutant is characterized by loss of $Mg^{2+}$ binding leading to loss of kinase activity. **(E)** Phosphorylation levels of ERK1/2 proteins in wild-type and BRAF -/- MEFs transduced with retrovirus expressing different forms of BRAF.

**Supplementary figure 2 references**

[1] Yang J, Cron P, Good VM, Thompson V, Hemmings BA, and Barford D: **Crystal structure of an activated Akt/protein kinase B ternary complex with GSK3-peptide and AMP-PNP.** *Nat Struct Biol* 2002, **9**(12): 940-944.

[2] Xu W, Doshi A, Lei M, Eck MJ, and Harrison SC: **Crystal structures of c-Src reveal features of its autoinhibitory mechanism.** *Mol Cell* 1999, **3**(5):629-638.

[3] Zhang X, Gureasko J, Shen K, Cole PA, and Kuriyan J: **An allosteric mechanism for activation of the kinase domain of epidermal growth factor receptor.** *Cell* 2006, **125**(6):1137-1149.

[4] Holm L and Rosenstrom P: **Dali server: conservation mapping in 3D.** *Nucleic Acids Res* 2010, **38**:W545-9. doi: 10.1093/nar/gkq366. Epub 2010 May 10.

[5] Haling JR, Sudhamsu J, Yen I, Sideris S, Sandoval W, Phung W, Bravo BJ, Giannetti AM, Peck A, Masselot A, Morales T, Smith D, Brandhuber BJ, Hymowitz SG, and Malek S: **Structure of the BRAF-MEK complex reveals a kinase activity independent role for BRAF in MAPK signaling.** *Cancer Cell* 2014, **26**(3):402-413.

[6] McRee DE: **XtalView/Xfit--A versatile program for manipulating atomic coordinates and electron density.** *J Struct Biol* 1999, **125**(2-3):156-165.

[7] Brunger AT, Adams PD, Clore GM, DeLano WL, Gros P, Grosse-Kunstleve RW, Jiang JS, Kuszewski J, Nilges M, Pannu NS, Read RJ, Rice LM, Simonson T, and Warren GL: **Crystallography & NMR system: A new software suite for macromolecular structure determination.** *Acta Crystallogr D Biol Crystallogr* 1998, **54**(Part 5): 905-921.

Supplementary figure 3

APC
Loss of heterozygosity; nt: A>T ; aa: R>X
Original allele frequency 0.67

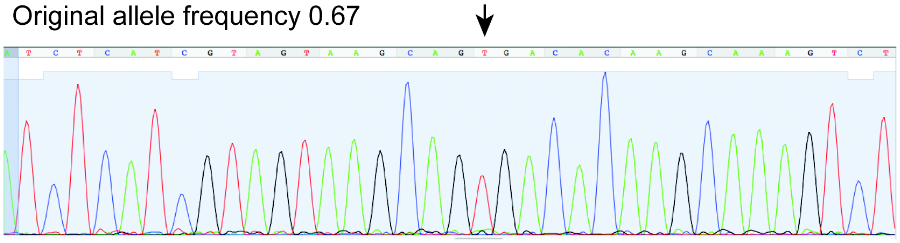

BRAF
Heterozygous; nt A>G; aa K>E
Original allele frequency 0.42

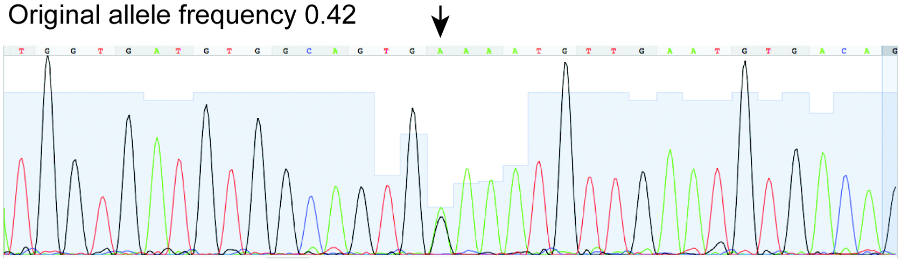

MAX
Heterozygous; nt G>A; aa R>Q
Original allele frequency 0.44

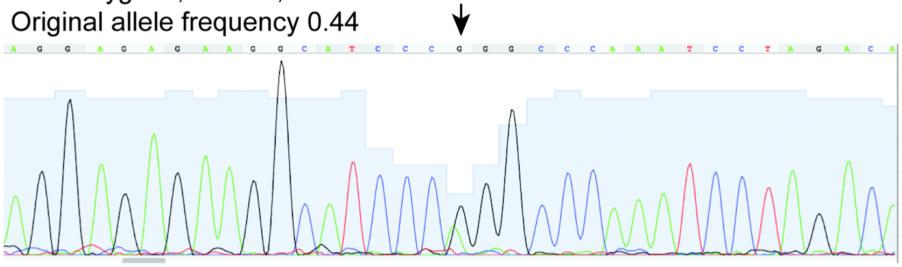

TP53
Loss of heterozygosity; nt G>A; aa R>Q
Original allele frequency 0.67

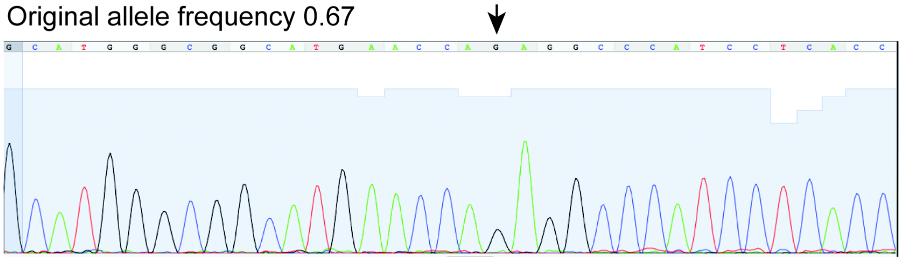

RPTOR
Heterozygous ; nt C>T; aa A>V
Original allele frequency 0.49

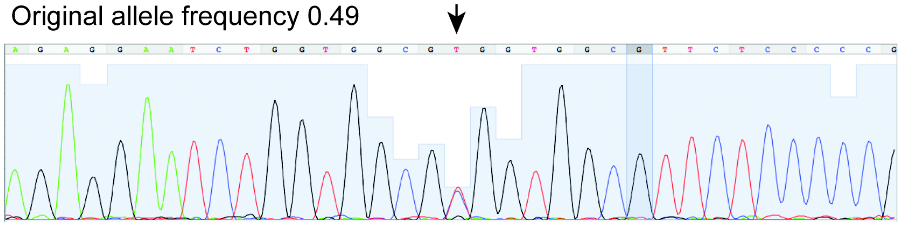

**Supplementary Figure 3:** Validation of patient-derived xenograft (PDX) tumor model by Sanger sequencing. PDX tumors recapitulate loss of heterozygosity and nucleotide mutations in *APC* and *TP53*, as well as heterozygous nucleotide mutations in *MAX*, *RPTOR*, and *BRAF* which were observed in the primary patient tumor. Tumors analyzed originated from 2$^{nd}$ generation tumors. Arrow denotes the point mutation and corresponding peak on the electropherogram. Primer sequences are available upon request.

Supplementary figure 4

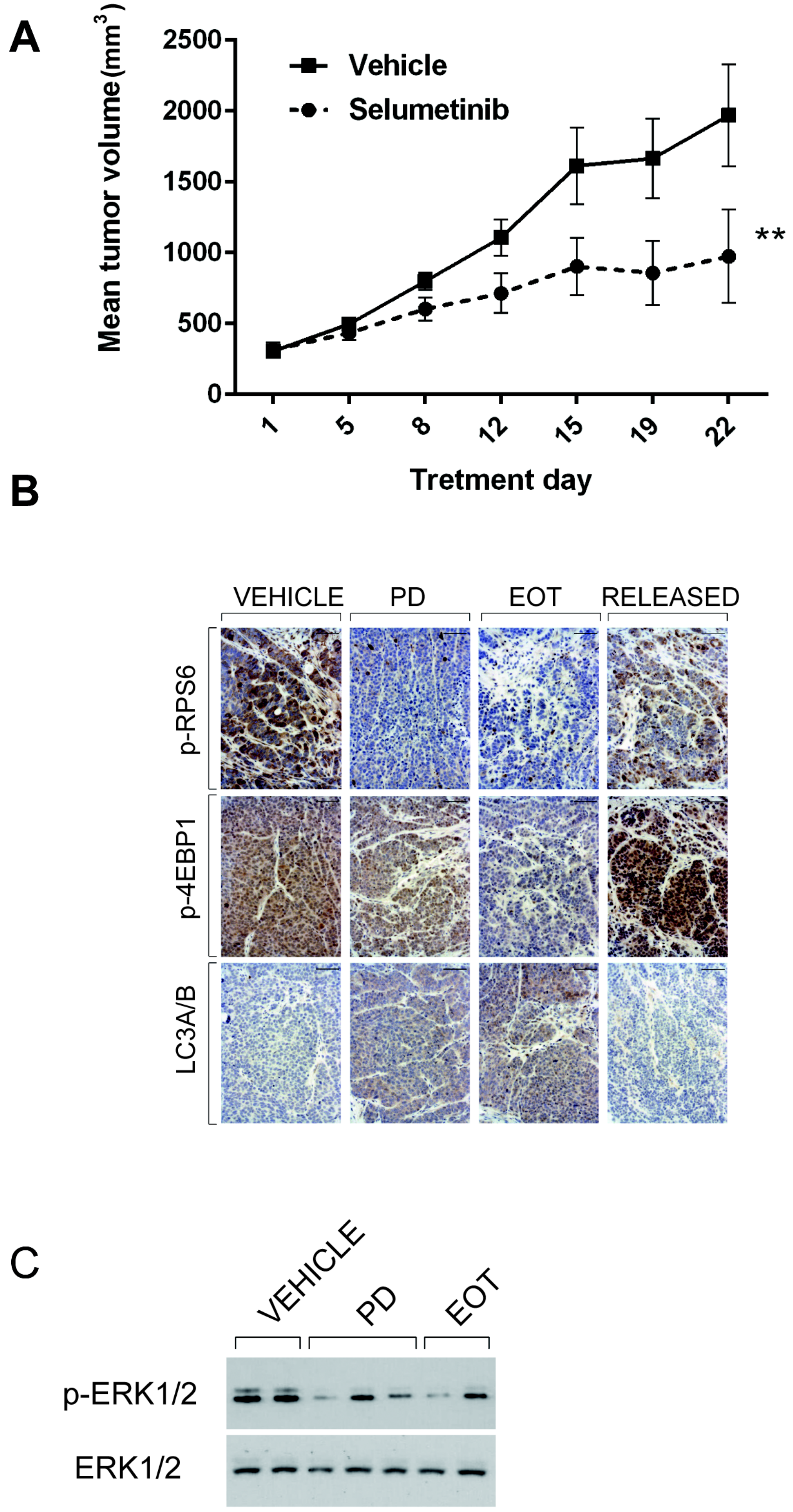

**Supplementary Figure 4: (A)** Tumor response to selumetinib. Mean and standard error of the mean are shown. ** $p<0.01$. **(B)** Representative immunohistochemical analysis showing inhibition of mTOR activity upon temsirolimus treatment. PD: pharmacodynamics. EOT: end of treatment. Scale bar = 100 µm. **(C)** Phosphorylation levels of ERK1/2 proteins in selumetinib-treated PDXs. PD: pharmacodynamics; EOT: end of treatment.
